# Augmentation of Bone Regeneration by Depletion of Stress-Induced Senescent Cells Using Catechin and Senolytics

**DOI:** 10.3390/ijms21124213

**Published:** 2020-06-13

**Authors:** Yoshitomo Honda, Anqi Huang, Tomonari Tanaka, Xiaoyu Han, Beiyuan Gao, Haitao Liu, Xinchen Wang, Jianxin Zhao, Yoshiya Hashimoto, Kazuyo Yamamoto, Naoyuki Matsumoto, Shunsuke Baba, Makoto Umeda

**Affiliations:** 1 Institute of Dental Research, Osaka Dental University, 8-1, Kuzuhahanazonocho, Hirakata, Osaka 573-1121, Japan; umeda-m@cc.osaka-dent.ac.jp; 2 Department of Oral Implantology, Osaka Dental University, 1-5-17, Otemae, Chuo-ku, Osaka 540-0008, Japan; huanganqident@gmail.com (A.H.); gao19940307@gmail.com (B.G.); baba-s@cc.osaka-dent.ac.jp (S.B.); 3 Graduate School of Science and Technology, Kyoto Institute of Technology, Matsugasaki, Sakyo-ku, Kyoto 606-8585, Japan; 4 Department of Operative Dentistry, Osaka Dental University, 1-5-17, Otemae, Chuo-ku, Osaka 540-0008, Japan; hanxy9308@gmail.com (X.H.); lh3562518@gmail.com (H.L.); yamamoto@cc.osaka-dent.ac.jp (K.Y.); 5 Department of Orthodontics, Osaka Dental University, 1-5-17, Otemae, Chuo-ku, Osaka 540-0008, Japan; cindyoops1126@hotmail.com (X.W.); jianxinzhao@hotmail.com (J.Z.); naoyuki@cc.osaka-dent.ac.jp (N.M.); 6 Department of Biomaterials, Osaka Dental University, 8-1, Kuzuhahanazonocho, Hirakata, Osaka 573-1121, Japan; yoshiya@cc.osaka-dent.ac.jp; 7 Department of Periodontology, Osaka Dental University, 1-5-17, Otemae, Chuo-ku, Osaka 540-0008, Japan

**Keywords:** cellular senescence, LPS, bone formation, catechin, EGCG, senolytics

## Abstract

Despite advances in bone regenerative medicine, the relationship between stress-induced premature senescence (SIPS) in cells and bone regeneration remains largely unknown. Herein, we demonstrated that the implantation of a lipopolysaccharide (LPS) sustained-release gelatin sponge (LS-G) increases the number of SIPS cells and that the elimination of these cells promotes bone formation in critical-sized bone defects in the rat calvaria. Histological (hematoxylin–eosin and SA-β-gal) and immunohistological (p16 and p21 for analyzing cellular senescence and 4-HNE for oxidation) staining was used to identify SIPS cells and elucidate the underlying mechanism. Bone formation in defects were analyzed using microcomputed tomography, one and four weeks after surgery. Parallel to LS-G implantation, local epigallocatechin gallate (EGCG) administration, and systemic senolytic (dasatinib and quercetin: D+Q) administration were used to eliminate SIPS cells. After LS-G implantation, SA-β-gal-, p16-, and p21-positive cells (SIPS cells) accumulated in the defects. However, treatment with LS-G+EGCG and LS-G+D+Q resulted in lower numbers of SIPS cells than that with LS-G in the defects, resulting in an augmentation of newly formed bone. We demonstrated that SIPS cells induced by sustained stimulation by LPS may play a deleterious role in bone formation. Controlling these cell numbers is a promising strategy to increase bone regeneration.

## 1. Introduction

Bone defects attributed to trauma, tumors, or inflammation are still a challenge in dentistry, orthopedics, and plastic surgery [1,2]. Several techniques using autogenous bone, biomaterials, and growth factors have been investigated for treatment of bone defects [3]. However, further research to elucidate fundamental mechanisms hindering osteogenesis is crucial to advance bone regenerative medicine.

All complex organisms are exposed to various stressors ex vivo and in vivo [4], including oxidative, mechanical, and osmotic stress; heat shock; radiation; and inflammatory cytokines. These stresses occasionally and irreversibly commit cell fate not only to apoptosis and oncogenesis, but also to cellular senescence, referred to as stress-induced premature senescence (SIPS) [4,5,6,7,8,9]. The phenotype of SIPS cells is generally defined by altered cell shape, irreversible cell cycle arrest, secretion of proinflammatory cytokines, and senescence-associated secretory phenotypes (SASP) [4]. Stress-induced cellular senescence is thought to be induced by DNA damage [4], and SIPS cells have similar phenotypes to cells undergoing replicative senescence [10]. Nevertheless, the roles of SIPS cells in vivo have only yet to be fully elucidated [11].

Lipopolysaccharide (LPS), a typical endotoxin present on the outer cell membrane of Gram-negative bacteria, is thought to be a potent contaminant in the medical field [12]. It is a well-known stimulant that potently activates immune systems and inflammation [13]. It can cause DNA damage in cells [14] and lead to formation of SIPS cells in vitro [15,16]. LPS stimulation potentially impairs bone healing [13]. Recently, we reported that the long-term presence of LPS in bone defects alters the induction of SIPS cells using two fabricated different LPS-gelatin sponges that released LPS at a distinct speed in vivo [17]. Compared with LPS rapid-release gelatin sponges, LPS sustained-release gelatin sponges (hereafter designated as LS-G) even with extremely low quantity of LPS generates more SIPS cells and causes less bone regeneration despite insignificant inflammation. Previous studies have reported that the elimination of senescent cells reduced bone loss in the INK-ATCC mouse model [18] and delayed wound healing in a p16-3MR mouse model [19]. Given these results, SIPS cells induced by sustained LPS stimulation may latently control bone regeneration. However, little is known about the role of these SIPS cells in bone regeneration.

**Figure 1:**
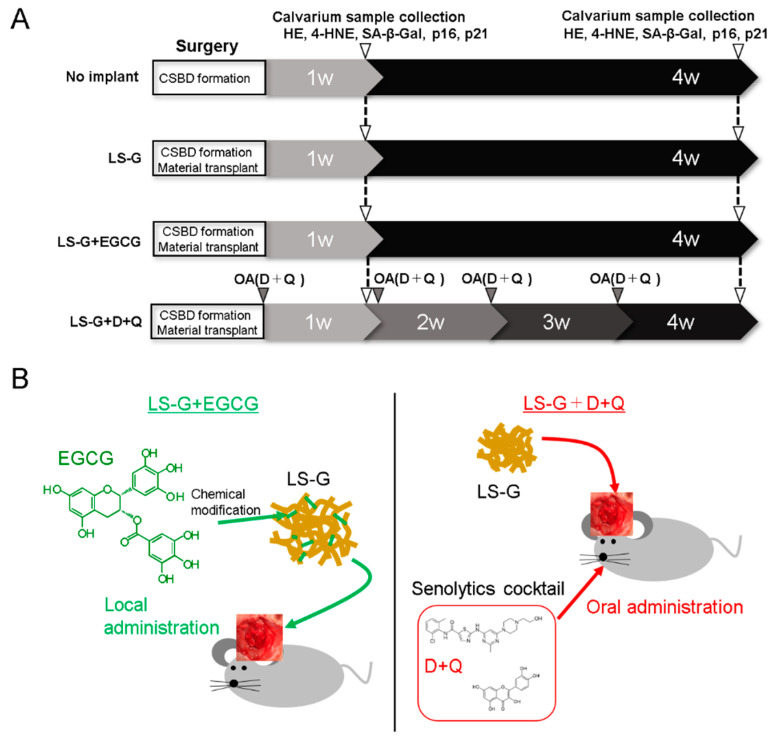
Flowchart of animal experiments: (**A**) workflow of animal experiments; and (**B**) schematic chart of LS-G+EGCG and LS-G+D+Q. CSBD, critical-sized bone defect; D, dasatinib; Q, quercetin; OA, oral administration; LS-G, implantation of lipopolysaccharide sustained-release gelatin sponges in bone defects; LS-G+EGCG, implantation of LS-G chemically modified with EGCG in bone defects; LS-G+D+Q, oral administration of senolytics (D+Q) with implantation of LS-G in bone defects.

To address these fundamental questions, this study was designed to elucidate the effect of elimination of SIPS cells on bone regeneration under the implantation of LS-G in critical-sized defects of rat calvaria. The rats were assigned to four treatment groups (Figure 1A). The two treatment groups to reduce the number of SIPS cells were as follows (Figure 1B): (1) local administration of catechin: the implantation of LS-G chemically modified with epigallocatechin gallate (EGCG) in defects (LS-G+EGCG); and (2) systemic administration of senolytics: the simultaneous implantation of LS-G alone in defects and oral administration of dasatinib (D: AdipoGen Life Sciences, Inc., San Diego, CA, USA) and quercetin (Q: FUJIFILM Wako Pure Chemical Co., Osaka, Japan) (LS-G+D+Q). D+Q was applied because the cocktail is widely used to eliminate senescent cells in vivo [20], although its function differs from that of EGCG.

## 2. Results

### 2.1. Characteristics of Sponges

**Figure 2:**
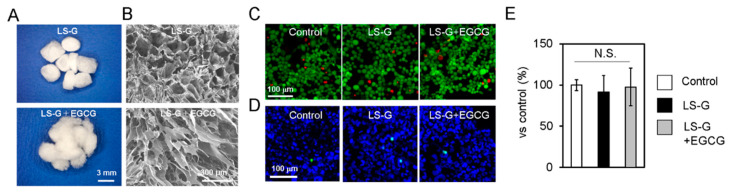
Characteristics of sponges. Macroscopic (**A**) and field-emission scanning electron microscopic (**B**) images of sponges. LS-G, LPS sustained-release gelatin sponges; LS-G+EGCG, LS-G chemically modified with EGCG. (**C**–**E**) Evaluation of cytotoxicity of LS-G and LS-G+EGCG in vitro. The osteoblastic cell line UMR106 was treated with or without the sponges for three days. Control, without the sponges. (**C**) Live and dead cell staining. Green, live cells; red, dead cells. (**D**) Apoptosis analysis. Green, apoptotic cells; blue, DAPI (4′,6-diamidino-2-phenylindole) staining representing nuclei. (**E**) Cell counting kit (CCK-8) colorimetric assay. Data are expressed as mean ± standard deviation (SD), *n* = 4. N.S., not significant.

LS-Gs containing 12.42 EU/mg of LPS were prepared as reported previously [17]. Our previous study revealed that LS-G could retain LPS in similar bone defects for at least three weeks. Both LS-G and LS-G+EGCG possessed irregular macropores (Figure 2A,B). Both LS-G and LS-G+EGCG showed no evidence of direct cytotoxic effects for ≤3 days in vitro (Figure 2C–E).

### 2.2. Hematoxylin–Eosin Staining for Bone Defects

**Figure 3:**
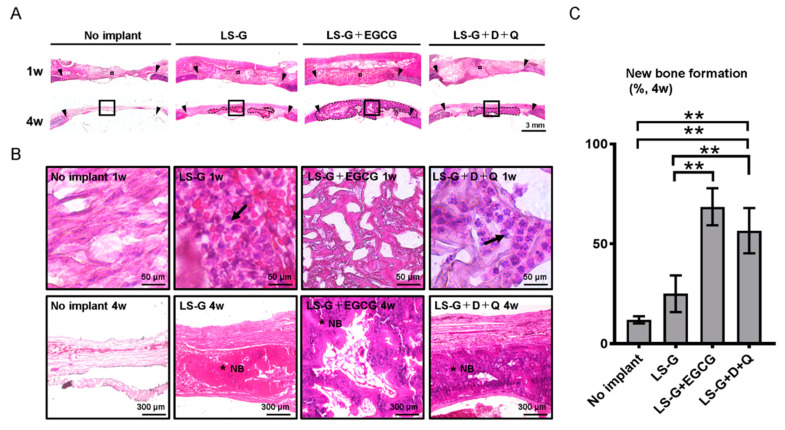
Histological evaluation of bone defects. Low (**A**) and high (**B**) magnification microscopic images of bone defects stained with hematoxylin–eosin one or four weeks after surgery. The image in (**B**) represents magnified squares in (**A**). Black triangles, edge of created bone defects. Black arrows, leucocytes. Black broken lines in (**A**) show newly formed bone. * NB, new bone; D, dasatinib; Q, quercetin. (**C**) Histomorphometric analysis of newly formed bones using the samples of H-E staining. Data are expressed as mean ± SD. ** *p* < 0.01. LS-G, implantation of LPS sustained-release gelatin sponges in bone defects; LS-G+EGCG, implantation of LS-G chemically modified with EGCG in bone defects; LS-G+D+Q, oral administration of senolytics (D+Q) with implantation of LS-G in bone defects.

To evaluate host reactions after surgery with or without the sponges, bone defects were stained with hematoxylin–eosin (H-E) (Figure 3). Several leucocytes in the defects were identified in the defect treated with LS-G and LS-G+D+Q one week after surgery, but not in the defects without LS-G (Figure 3B). Fewer leucocytes were detected in the defects treated with LS-G+EGCG.

### 2.3. Increase in SIPS Cell after LS-G Implantation

**Figure 4:**
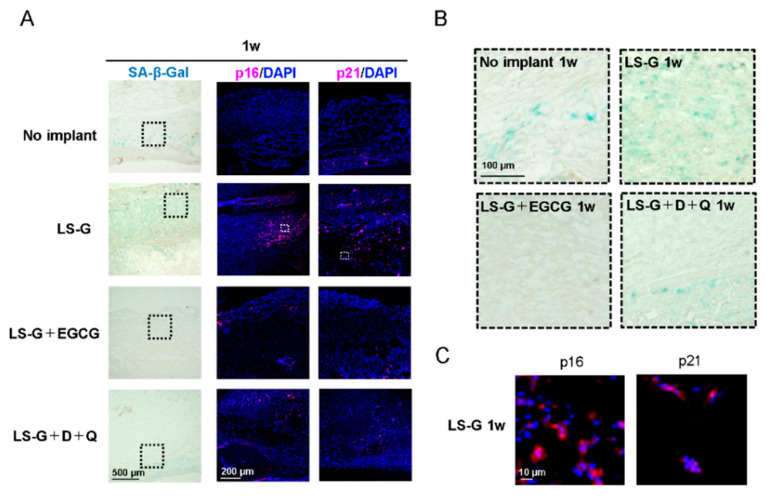
Detection of cellular senescence in bone defects one week after surgery. Low (**A**) and high (**B**) magnification microscopic images of bone defects stained with SA-β-gal or with antibodies for p16 and p21. (**C**) Representative magnified images of senescent cells induced by LS-G. Broken lines in (**A**) show magnified area for (**B**). White squares in (**A**) show magnified area for (**C**). D, Dasatinib; Q, Quercetin; LS-G, implantation of LPS-sustained gelatin sponges in bone defects; LS-G+EGCG, implantation of LS-Gs chemically modified with EGCG in bone defects; LS-G+D+Q, oral administration of D+Q with implantation of LS-Gs in bone defects.

**Figure 5:**
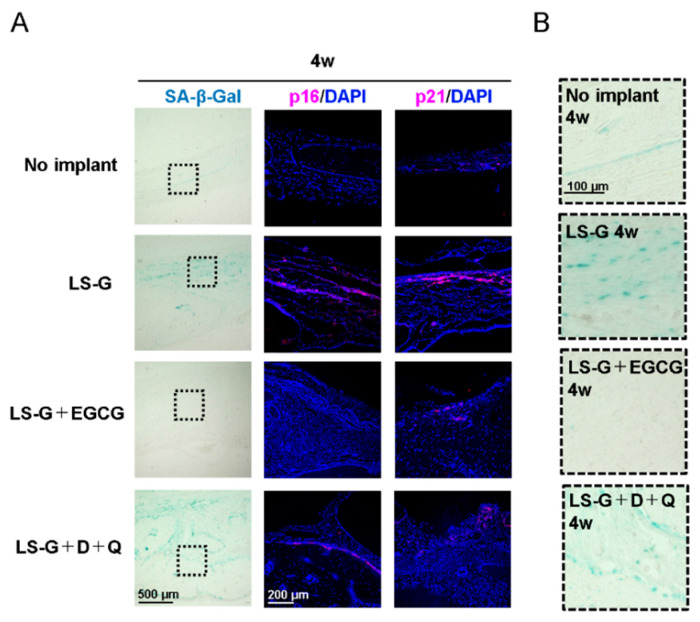
Detection of cellular senescence in bone defects four weeks after surgery. Low (**A**) and high (**B**) magnification microscopic images of bone defects stained with SA-β-gal or antibodies for p16 and p21. Broken lines in (**A**) show magnified area for (**B**). D, Dasatinib; Q, Quercetin; LS-G, implantation of LPS-sustained gelatin sponges in bone defects; LS-G+EGCG, implantation of LS-Gs chemically modified with EGCG in bone defects; LS-G+D+Q, oral administration of D+Q with implantation of LS-Gs in bone defects.

Although a universal marker solely expressed in senescent cells has not been identified, SA-β-gal, p16, and p21 are widely used biomarkers to determine cellular senescence [11,19]. To identify senescent cells in bone defects, histological staining with SA-β-gal and immunofluorescence staining with p16 and p21 antibodies were performed one (Figure 4) and four weeks (Figure 5) after surgery on the defects. The staining levels of SA-β-gal, p16, and/or p21 in defects treated with LS-G remained strong for up to four weeks (Figure 4; Figure 5) after surgery. Both EGCG and D+Q apparently attenuated the staining levels of p16 and p21 in the defects for up to four weeks. Our results suggest that the implantation of LS-G induced SIPS cells, while local and systemic administration of EGCG and D+Q, respectively, successfully reduced SIPS in bone defects.

### 2.4. Histomorphometric Analysis of Newly Formed Bone

**Figure 6:**
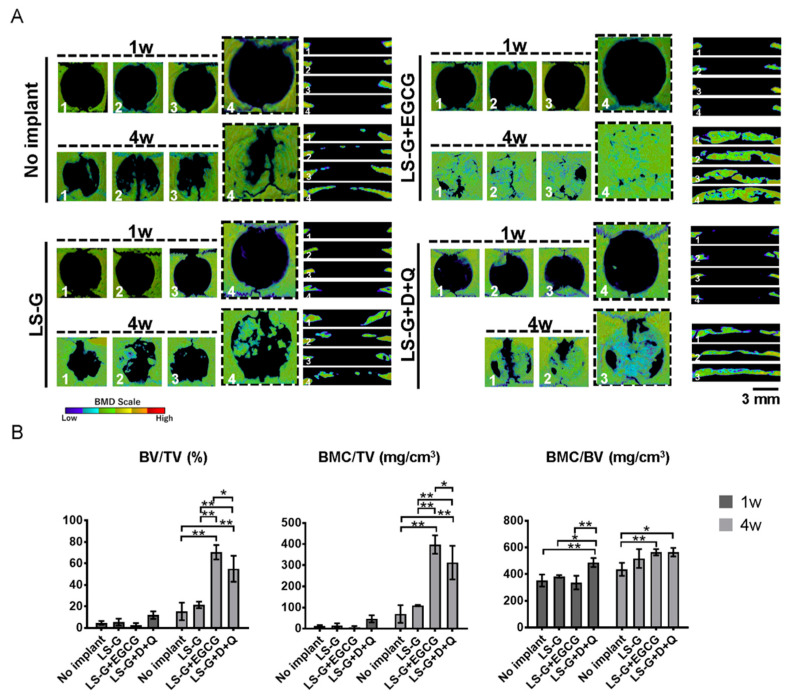
Histomorphometric analysis using microcomputed tomography (μCT). (**A**) Vertical and lateral views of bone mineral density images of bone defects one or four weeks after surgery. Four defects were analyzed in each group. One rat treated with LS-G+D+Q for four weeks unexpectedly died at three weeks during the experimental period. (**B**) Morphometric analysis using μCT. BV, bone volume; TV, total volume; BMC, bone mineral content; D, Dasatinib; Q, Quercetin; LS-G, implantation of LPS-sustained gelatin sponges in bone defects; LS-G+EGCG, implantation of LS-Gs chemically modified with EGCG in bone defects; LS-G+D+Q, oral administration of D+Q with implantation of LS-Gs in bone defects. Data are expressed as mean ± SD. * *p* < 0.05, ** *p* < 0.01.

To confirm whether the elimination of senescent cells from bone defects altered bone regeneration, we histomorphometrically analyzed bone defects using microcomputed tomography (μCT) and H-E staining (Figure 3; Figure 6). The use of both EGCG and D+Q markedly elevated the radiopacity of bone defects (Figure 6A,B). H-E staining showed that the high radiopacity was the newly formed bone (Figure 3B,C).

### 2.5. Oxidation in the Bone Defects

**Figure 7:**
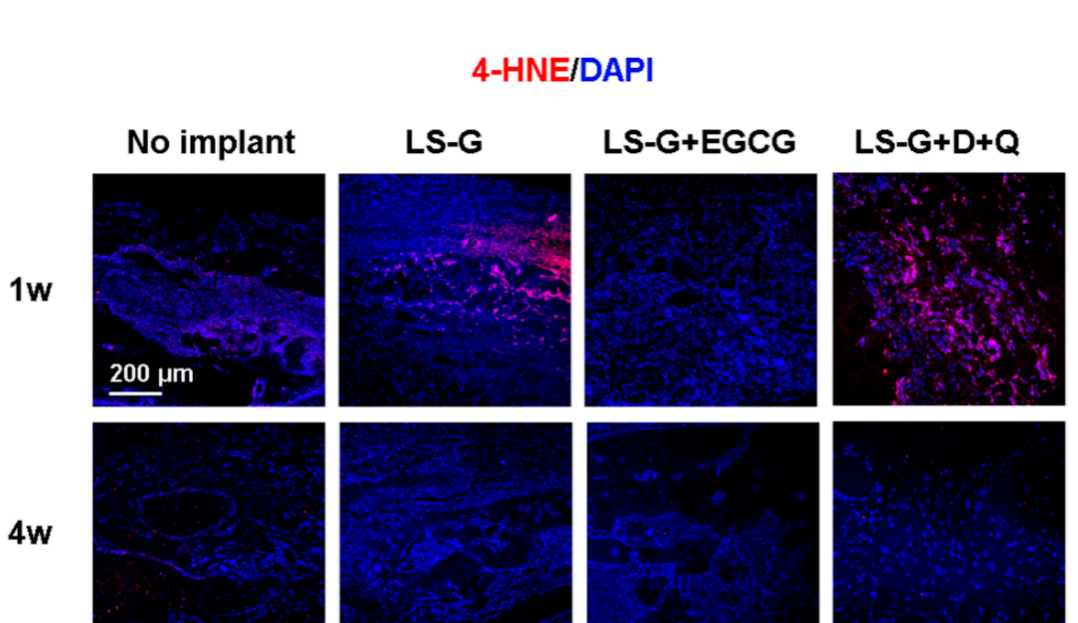
Evaluation of oxidation in bone defects one and four weeks after surgery. Bone defects stained with 4-HNE antibodies (for detecting oxidation). D, Dasatinib; Q, Quercetin; LS-G, implantation of LPS-sustained gelatin sponges in bone defects; LS-G+EGCG, implantation of LS-Gs chemically modified with EGCG in bone defects; LS-G+D+Q, oral administration of D+Q with implantation of LS-Gs in bone defects.

Oxidative stress plays a significant role in DNA damage, potentially initiating cellular senescence [9,21]. To confirm the detailed mechanisms underlying the reduction of senescent cells by EGCG and D+Q, we performed immunofluorescence staining of the defects using antibodies for 4-HNE (one and four weeks after surgery (Figure 7)), which is a biomarker for identifying oxidation in tissue [22]. The defects treated with LS-G alone and LS-G+D+Q showed strong 4-HNE staining one week after implantation (Figure 7). Administration of EGCG significantly reduced the staining levels of 4-HNE, but administration of D+Q did not. Approximately four weeks after surgery, the staining level of 4-HNE was similar in all the defects.

## 3. Discussion

In this study, we demonstrated that implantation of LS-G induced SIPS cells in critical-sized bone defects in rat calvaria. The use of EGCG locally or D+Q systemically attenuated the development of SIPS cells in the defects. This decrease in SIPS cells effectively enhanced bone formation by LS-G for ≤4 weeks.

Thus far, few studies have reported that gelatin potentially induces inflammatory reactions in host tissues [23], while the early inflammatory reaction is augmented after implantation of hydrogel containing a reagent-grade gelatin [24]. Zhao et al. showed that proinflammatory cytokine production increased in the presence of both gelatin and NF-κB activation [23]. This discrepancy might be due to the difference in the bonding between LPS and gelatin. Recently, we identified that reagent-grade gelatin contains a small amount of LPS [17]. We fabricated vacuum-heated gelatin sponges (LS-G) [17] because dehydrothermal treatment using vacuum heating is known to enhance ester bonding between carboxyl and hydroxyl groups of molecules [25]. The vacuum heating technique has successfully enabled LS-Gs to sustain the release of LPS, thereby inducing more cellular senescence in defects for three weeks than that by gelatin sponge lacking ester bonding between LPS and gelatin [17]. The amount of LPS used in the LS-G in defects (28.98 pg per defect) is far lower than the LD_50_ (3 mg/kg) [26]. LS-G contains 345–17,253 times less LPS than other doses applied previously in vivo [27,28,29]. To confirm the reproducibility of our previous study, we used same the LS-Gs, the material that could result in numerous SIPS cells in defects for four weeks. The results support the evidence that the use of dehydrothermal treatments causing physical cross-linking between microbial components and materials is a usable technique to fabricate experimental models of cellular senescence and chronic inflammation.

Two different techniques to eliminate SIPS cells (Figure 1B) were adopted in the present study because: (1) EGCG isolated from green tea is a well-known polyphenol, which has anti-inflammatory [30] and anti-oxidant properties [31]. It has also been reported to suppress cellular senescence in vitro [32,33]. Recent studies showed that the chemical modification of gelatin with EGCG elicits greater pharmacological effect and bone formation than a simple mix of EGCG and gelatin [34,35]. Therefore, we hypothesized that the local administration of EGCG would also suppress SIPS cells in vivo. (2) Oral administration of D+Q directly successfully reduces the number of senescent cells in vivo [20]. Moreover, various studies have validated the function of D+Q as a senolytic in the fields of longevity, arteriosclerosis, obesity-related metabolic disfunction, and bone loss, among others [18,20,36,37]. As can be seen in Figure 4 and Figure 5, the two techniques attenuated the generation of SIPS cells induced by LS-G implantation up to four weeks.

SA-β-gal is a common biomarker for detecting cellular senescence [11], but its reliability in bone biology is still controversial [38]. Senescent cells and hyperfunctional macrophages are known to be positive for SA-β-gal staining [39], as β-gal staining reflects hyperfunctional lysosomes [40]. Therefore, we confirmed the presence of SIPS with SA-β-gal staining and the well-known senescence biomarkers, p16 and p21. The staining levels of p16 or p21 were approximately equal to those of SA-β-gal in the defects.

LPS causes inflammation and generates reactive oxygen species, resulting in oxidative stress [41]. Oxidative stress promotes cellular senescence [4]. In our in vivo study, early inflammatory reactions augmented by LS-G were distinguishable by the presence of leucocytes in the sponges one week after implantation; however, this effect was attenuated by Week 4. Coincident with this inflammatory reaction, the staining levels of 4-HNE increased at one week but not at four weeks. Meanwhile, although LS-G significantly increased SIPS cells in the defects after one week, these SIPS remained in the defects for four weeks. Additionally, we determined that the used sponges caused negligible cytotoxic effects on the osteoblastic cell line UMR106 in vitro (Figure 2C–E). These results suggest that, although the inflammatory reaction that caused oxidation may be temporally and partially associated with the induction of SIPS cells at early stage, its effect was limited. Direct feeble and lasting stimulation by residual LPS of LS-G is likely to cause cellular senescence.

The staining levels of 4-HNE in the defects indicated that the EGCG and D+Q treatments caused different host reactions: EGCG reduced oxidation after one week, but D+Q did not (Figure 7). Although excessive oxidation is known to attenuate osteogenesis [42], there was no strong attenuation of bone formation in defects treated with LS-G+D+Q (Figure 6). Both LS-G+EGCG and LS-G+D+Q could induce superior bone regeneration compared with LS-G alone. Simultaneously, both treatments successfully reduced the staining levels of SA-β-gal, p16, and p21 after sponge implantation. These results suggest that SIPS cells rather than direct oxidation stress are likely to play a deleterious role in bone regeneration in defects.

In the present study, we could partially verify the relation between SIPS cells induced by LPS stimulation and bone regeneration using a rat calvarium model. However, our study had several limitations. Details regarding the type of senescent cells that are present in or around the sponges are still unknown. It is possible that the species and age of animals as well as the implantation sites alter these reactions. Moreover, we could not determine the exact role of the SASP from induced SIPS cells in the bone formation and remodeling processes. Additional experiments would therefore be essential to deepen the understanding of the mechanisms underlying the relationship between LPS-induced SIPS cells and bone regeneration in vivo. However, our report does provide insights for the advancement of bone regenerative medicine.

## 4. Materials and Methods

### 4.1. Characterization of the Sponges

Reagent-grade type A gelatin from pig skin (Cat. No. G2500) containing LPS was purchased from Sigma-Aldrich (St. Louis, MO, USA). LS-G and LS-G+EGCG were prepared as described before [17]. To produce LS-G, 100 mg gelatin were dissolved in 10 mL MilliQ water at 70 °C. The resulting solution was frozen and freeze-dried using DC800 (Yamato Co., Ltd., Tokyo, Japan) in φ 5 mm silicon tubes. The prepared gelatin sponges were then vacuum-heated at 150 °C for 24 h and a gauge pressure of −0.1 MPa to promote physical cross-linking using ETTAS AVO-250NS (AS ONE, Osaka, Japan). LPS level in sponges was evaluated utilizing a ToxinSensor Chromogenic LAL Endotoxin Assay Kit (L00350, GenScript Biotech Inc., Piscataway, NJ, USA) according to the manufacturer’s instructions, as in our previous study [17]. We previously demonstrated that LS-G could retain LPS for at least three weeks in a similar bone defect model to the one used in this study [17]. For LS-G+EGCG, an aqueous synthesis method was applied before lyophilization and vacuum heating. Briefly, 100 mg gelatin, 0.07 mg EGCG, 69.2 mg 4-(4,6-dimethoxy-1,3,5-triazin-2-yl)-4-methylmorpholinium chloride (DMT-MM), and 27.5 μL N-methylmorpholine (NMM) were dissolved and mixed in 5 mL MilliQ water at 23 °C. The aforementioned EGCG dose was selected based on our previous results of superior bone formation (pharmacological effect) [43]. After dialysis to remove the residual reagents using Spectra/Por7 MWCO 1000 (Spectrum Labs, CA, USA) in water in the dark, the resulting solution was diluted to 10 mL with MilliQ water, frozen, lyophilized, and vacuum-heated. The sponges were vacuum-heated at 150 °C for 24 h. The morphology of the sponges was analyzed using a stereomicroscope (SZX12, Olympus Inc., Tokyo, Japan) and field-emission scanning electron microscope (FE-SEM, S-4800; Hitachi, Tokyo, Japan).

### 4.2. Cytotoxic Effect of the Sponges

Rat osteoblastic cell line UMR106 (CRL-1661, American Type Culture Collection, Manassas, VA, USA) was seeded in 48-well plates at a density of 1 × 10^4^/well and cultured in Dulbecco’s modified Eagle’s medium with 10% fetal bovine serum and 1% antibiotics. One day after cell seeding, the cells were treated with or without 0.5 mg of sponges in each well for three days. The wells without sponges were considered negative control. The cytotoxic effect of the sponges was estimated using LIVE/DEAD Viability/Cytotoxicity Kit (Molecular Probes, Eugene, OR, USA), CellEvent Caspase-3/7 Green Detection Reagent (Thermo Fisher Scientific, Waltham, MA, USA) with DAPI Fluoromount-G (4′,6-diamidino-2-phenylindole; SouthernBiotech, Birmingham, AL, USA), and a cell counting kit (CCK-8) colorimetric assay (Dojindo Molecular Technologies, Kumamoto, Japan) according to the manufacturer’s instructions.

### 4.3. Animal Experiments

As senescent cells accumulate with age [44], eight-week-old Sprague-Dawley rats were used to minimize the misidentification of age-related senescent cells as SIPS cells. The animal experiments in this study were conducted with the permission of and in accordance with the guidelines approved by the local ethics committee of Osaka Dental University (Approval No. 19-04002, 27 May 2019). The male Sprague-Dawley rats were obtained from SHIMIZU Laboratory Supplies Co. (Kyoto, Japan). The rats were housed in a ventilated room with a 12 h light/dark cycle during the experiments. At the center of their calvaria, 9-mm-sized bone defects, commonly recognized as the critical size defect, were created using a trephine bar as reported previously [45]. During surgery, the rats were anesthetized with an intraperitoneal injection of a mixture of medetomidine hydrochloride (0.15 mg/kg; Domitor; Zenoaq, Fukushima, Japan), midazolam (2 mg/kg; Midazolam Sandoz, Sandoz KK, Yamagata, Japan), and butorphanol tartrate (2.5 mg/kg; Vetorphale, Meiji Seika Parma Co., Ltd., Tokyo, Japan). They were divided into four groups with four rats per group (Figure 1A): Group 1 (control): no implant; Group 2 (control): implantation of LS-G; Group 3: implantation of LS-G+EGCG; and Group 4 (LS-G+D+Q): oral administration of D+Q and implantation of LS-G. One rat treated with LS-G+D+Q for four weeks unexpectedly died at three weeks during the experimental period. D and Q were diluted at a ratio of 1:10 in 10% PEG400 in water (Cat. No. 25322-68-3, FUJIFILM Wako Pure Chemical Co., Osaka, Japan) and orally administered by gavage once a week at 5 (D) and 50 mg/kg (Q). Ten sponges (one sponge: 3 × 3 mm, D × H) were implanted in each defect. We used two different control groups in view of the distinct processes of bone formation and SIPS induction. The no implant group (without gelatin and LPS), which basically lacked strong bone forming ability and the ability for SIPS induction, was used as a criterium to confirm the augmentation of both bone formation and SIPS cell induction by other groups. LS-G (with gelatin and LPS), which has the potential to induce bone formation and SIPS cells, was used to confirm the effect of elimination of SIPS cells on bone regeneration.

### 4.4. Histomorphometric Analysis

At one and four weeks after surgery, the rats were euthanized using isoflurane. The calvaria was retrieved and fixed with 4% paraformaldehyde in 0.1 M phosphate buffer for histological and morphometric analyses described below. Bone formation in bone defects was quantified using μCT analysis (SMX-130CT; Shimadzu, Kyoto, Japan or inspeXio SMX-225CT; Shimadzu) and histological staining. Each sample was scanned at 55 kV and 90 μA using a voxel size of 30 × 30 × 30 μm^3^. The 3D morphology of the calvaria was reconstructed using TRI/3D bone software (RATOC Systems Engineering Co., Ltd., Tokyo, Japan). To estimate bone formation and quality in bone defects, the following parameters were quantified: bone volume (BV)/total volume (TV); bone mineral content (BMC)/TV; and BMC/BV. Cylindrical phantoms containing hydroxyapatite (200–1550 mg/cm^3^) were used to calculate the BMC representing calcified bone tissue.

### 4.5. Histological and Immunofluorescent Staining

After scanning calvaria with μCT, 4 μm-thick, non-decalcified sections were prepared from each sample using the Kawamoto method [46] and a cryotome (Leica CM3050S; Leica Biosystems Inc., Buffalo Grove, IL, USA) for histological and immunohistological observation. The sections were stained with H-E to estimate the presence of newly formed bone or leucocytes (inflammatory response) using HS All-in-one Fluorescence Microscope (BZ-9000, Keyence, Osaka, Japan). Quantitative calculations of newly formed bone were carried out using Photoshop CC2019 (Adobe Systems Inc., San Jose, CA, USA) and NIH ImageJ version 1.52a (Bethesda, MD, USA). The percentage of the newly formed bone in the defect was calculated as follows: (newly formed bone area/total tissue area in defect) × 100.

To evaluate SIPS, sections were stained with SA-β-gal using Senescence Detection Kit (Cat. No.: ab65351, Abcam, Cambridge, UK) according to the manufacturer’s instruction and analyzed with BZ-9000 (Keyence). For immunofluorescence staining, the sections were stained with primary antibodies as follows: anti-4-HNE polyclonal antibodies conjugated with Alexa Fluor 647 (Cat. No. bs-6313r-a647; Bioss Inc., MA, USA; 1:200), anti- p21 (Cat. No. 10355-1-AP; ProteinTech Group, Inc., Rosemont, IL, USA; 1:200), and anti-p16-INK4A (Cat. No. 10883-1-AP; ProteinTech Group Inc.; 1:200). We used anti-IgG(H+L) antibodies conjugated with Alexa Fluor (Cat. No. ab150079; Life Technologies, Carlsbad, CA, USA; 1:100, 647 nm) as secondary antibodies. After washing with phosphate-buffered saline, the sections were mounted with DAPI Fluoromount-G (SouthernBiotech) and analyzed using ZEISS LSM700 (Carl Zeiss Microscopy, Jena, Germany).

### 4.6. Statistical Analysis

Statistical significance of mean values was assessed using GraphPad Prism 8 (GraphPad Software Inc., San Diego, CA, USA) and one-way analysis of variance, followed by Tukey–Kramer test to determine significance.

## 5. Conclusions

In this study, we demonstrated that the reduction in the number of SIPS cells using two different techniques enhanced bone regeneration. Our results suggest that SIPS cells are at least partially associated with the modulation of bone regeneration. These data possibly provide basic knowledge for the development of promising new biomaterials and bone regeneration therapies.

## Abbreviations

LPS	lipopolysaccharide
LS-G	LPS sustained-release gelatin sponge
EGCG	epigallocatechin gallate
D+Q	dasatinib and quercetin
CBSD	critical-sized bone defect
OA	oral administration
